# Application of Convolution Neural Network Algorithm Based on Multicenter ABUS Images in Breast Lesion Detection

**DOI:** 10.3389/fonc.2022.938413

**Published:** 2022-07-04

**Authors:** Jianxing Zhang, Xing Tao, Yanhui Jiang, Xiaoxi Wu, Dan Yan, Wen Xue, Shulian Zhuang, Ling Chen, Liangping Luo, Dong Ni

**Affiliations:** ^1^Department of Medical Imaging Center, The First Affiliated Hospital, Jinan University, Guangzhou, China; ^2^Department of Ultrasound, Remote Consultation Center of ABUS, The Second Affiliated Hospital, Guangzhou University of Chinese Medicine, Guangzhou, China; ^3^Medical Ultrasound Image Computing Lab, Shenzhen University, Shenzhen, China

**Keywords:** automatic breast ultrasound (ABUS), convolution neural network, breast cancer, detection, validation data

## Abstract

**Objective:**

This study aimed to evaluate a convolution neural network algorithm for breast lesion detection with multi-center ABUS image data developed based on ABUS image and Yolo v5.

**Methods:**

A total of 741 cases with 2,538 volume data of ABUS examinations were analyzed, which were recruited from 7 hospitals between October 2016 and December 2020. A total of 452 volume data of 413 cases were used as internal validation data, and 2,086 volume data from 328 cases were used as external validation data. There were 1,178 breast lesions in 413 patients (161 malignant and 1,017 benign) and 1,936 lesions in 328 patients (57 malignant and 1,879 benign). The efficiency and accuracy of the algorithm were analyzed in detecting lesions with different allowable false positive values and lesion sizes, and the differences were compared and analyzed, which included the various indicators in internal validation and external validation data.

**Results:**

The study found that the algorithm had high sensitivity for all categories of lesions, even when using internal or external validation data. The overall detection rate of the algorithm was as high as 78.1 and 71.2% in the internal and external validation sets, respectively. The algorithm could detect more lesions with increasing nodule size (87.4% in ≥10 mm lesions but less than 50% in <10 mm). The detection rate of BI-RADS 4/5 lesions was higher than that of BI-RADS 3 or 2 (96.5% vs 79.7% vs 74.7% internal, 95.8% vs 74.7% vs 88.4% external). Furthermore, the detection performance was better for malignant nodules than benign (98.1% vs 74.9% internal, 98.2% vs 70.4% external).

**Conclusions:**

This algorithm showed good detection efficiency in the internal and external validation sets, especially for category 4/5 lesions and malignant lesions. However, there are still some deficiencies in detecting category 2 and 3 lesions and lesions smaller than 10 mm.

## Introduction

Breast cancer is the most common cancer and a leading cause of cancer death in women worldwide, but precise detection can provide an opportunity for timely treatment (1). Among the various detection methods, B-mode ultrasound screening technology is favored and recommended as a routine diagnostic tool because of its low cost and rapid imaging (2). Although breast ultrasound imaging can characterize the suspicious tumor area of the breast tissue, it has high technical dependence on the operator, poor diagnostic repeatability, long time-consuming and low accuracy, and massive daily image analysis aggravates the burden of clinical radiologists (3). Furthermore, the inconsistency of different radiologists on the same image may lead to severe false-positive problems, thereby delaying effective treatments (4).

Automatic breast ultrasound (ABUS) imaging has become an essential tool in breast cancer diagnosis. ABUS is considered to have high repeatability, low operator dependence, less time consuming by radiologists in image acquisition, automatic three-dimensional reconstruction of the whole breast, coronal information, and a relatively wide observation field. Studies have shown that mammography (MG) plus ABUS examination can increase the detection rate of breast cancer in women with dense breasts, particularly the detection rate of small lesions (5). A multi-center study on Chinese women showed that ABUS had good reliability compared with handhold ultrasound (HHUS) and MG (6). The other study conducted in the United States showed that it could help improve the detection rate of breast cancer by adding ABUS to breast cancer screening (7).

Although ABUS has many advantages, it also inevitably aggravates the workload of screening and diagnostic examiners. Different computer-aided diagnosis (CAD) systems have been developed to standardize and accelerate diagnostic procedures (8). Relevant studies suggest that computer-aided diagnosis software could effectively improve the detection of lesions and the speed of diagnosis (9). However, in breast cancer imaging research, deep learning (AI or computer-aided diagnosis CAD) has mainly focused on mammography or ultrasound 2D/3D imaging combined with deep learning (10–12). In lesion detection and diagnosis, accurate segmentation of the breast mass in a 3D ABUS image is an essential task in ABUS image analysis. It also plays a vital role in designing a computer-aided detection or diagnostic system (13–15). Recently, deep learning techniques have made significant progress in medical image segmentation (16–18). The convolution neural network (CNN) has become a promising choice in breast ultrasound image segmentation (19–22).

The diagnostic process of breast lesions includes detection, diagnosis, and treatment. Lesion detection is the premise of this diagnosis. A single ABUS volume image could have 320 frames with a layer thickness of 0.5 mm. The amount of data in ABUS images is more than that in most natural images. The cost of manually marking breast lumps is high. Direct training of large-scale segmentation networks with tens of millions of parameters may introduce potential over-fitting (23). This study aimed to evaluate a convolution neural network algorithm for breast disease detection, developed based on ABUS image and Yolo v5. The efficiency and accuracy of the algorithm were analyzed in detecting lesions with different allowable false positive values and lesion sizes, and the differences were compared and analyzed, which included the various indicators in internal validation and external validation data.

## Materials and Methods

### Patients

This multi-center retrospective study was conducted following the Declaration of Helsinki and was approved by the local institutional review board (ZE2020-232). A total of 32,493 cases of ABUS examination were analyzed, which were collected from 7 hospitals (GDHCM, BHLY, CFFIC, PHHKS, YDHSZ, LKMC, and JBMH) between October 2016 and December 2020. The inclusion criteria of this study were: ① The quality control requirements of ABUS were met; ② Malignant lesions were confirmed by pathology; ③ The clinical information of the case was complete; and ④ benign lesions required a biopsy or follow-up for more than 2 years by ultrasound, mammography or MRI. The exclusion criteria in this study were: ① the quality control requirements of the image were not met; ② the patient had a history of breast trauma, surgery, mastitis, etc.; ③ suspected malignant tumor without pathological results; ④ benign lesions failed to follow up as required; and ⑤ other conditions could affect diagnosis. Finally, 30,515 cases were excluded from the study.

All training and test data were from the Guangdong Province Hospital of Chinese Medicine because each case needed a bilateral breast examination, and some lesions could be displayed in two or three-volume data. The training set collected 3,457 ABUS volume data from 936 cases (age range 18–70 years, average 41.54 ± 11.22 years), 221 malignant lesions and 3,662 benign lesions, and 791 confirmed lesions by pathology. Another 1,406 ABUS volume data of 301 cases (age range 26–69 years, average 42.37 ± 12.48 years) were used for validation, including 57 malignant lesions and 885 benign lesions, and 247 lesions were confirmed by pathology.

**Figure 1:**
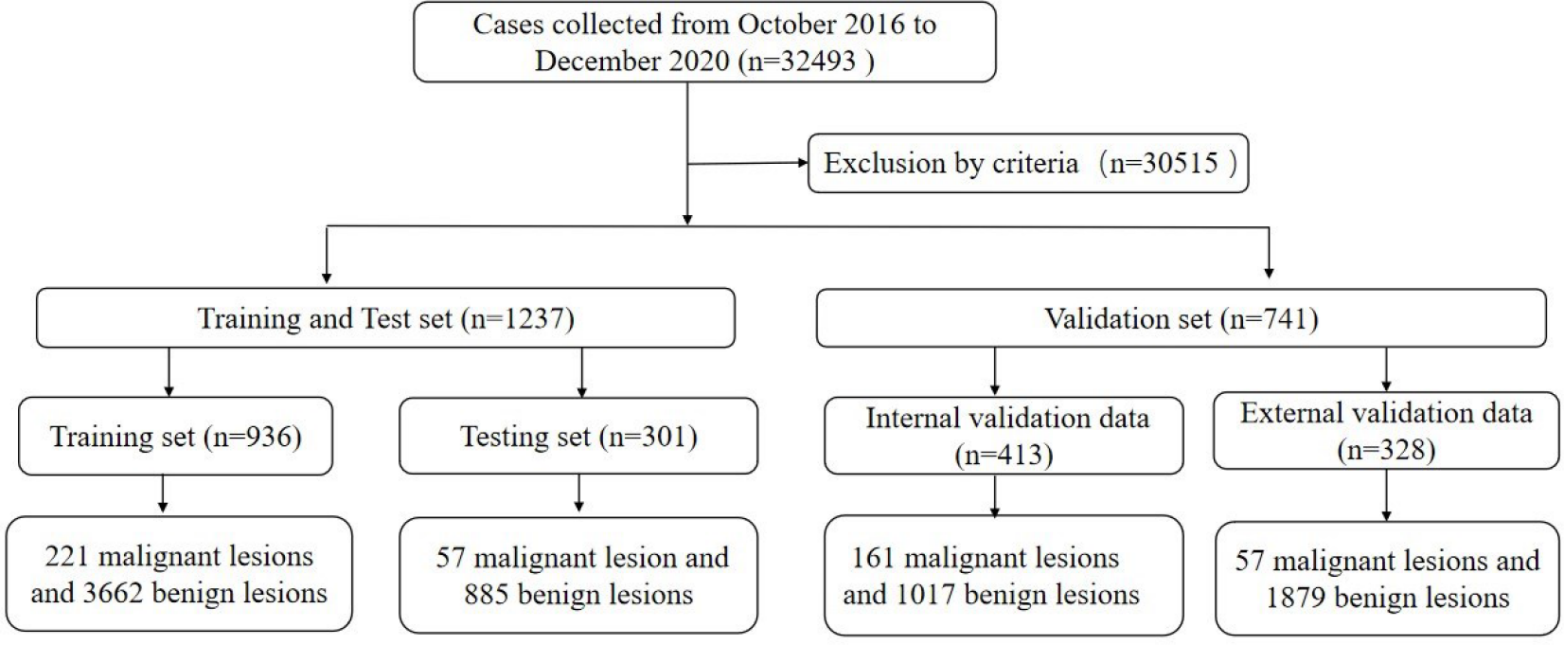
Flowchart of the study population in the training set, testing set and validation set.

The other 741 cases with 2,538 volume data were included in the validation data. A total of 452 volume data of 413 cases (age range 23–67 years, average 41.21 ± 11.93 years) were used as internal validation data (IVD), including 161 malignant lesions and 1,017 benign lesions. A total of 214 lesions of BI-RADS 4 or 5 and 47 lesions of BI-RADS 3 were confirmed by pathology. The other lesions were followed up to rule out the possibility of malignancy. A total of 328 cases (age range 22–65 years, average 40.32 ± 13.44 years) with 1,086 volume data were used as external validation data (EVD), which included 57 malignant lesions and 1,879 benign lesions. A total of 96 lesions of BI-RADS 4 or 5 and 29 lesions of BI-RADS 3 were confirmed by pathology, and the other lesions were followed up as required. All cases with dense breasts were collected in this study (**Figure 1**).

### Quality Control and Reference Standard

All cases were scanned according to the scanning specifications recommended by the ABUS use manual. The standard images of unilateral breast scanning are AP, LAT, and MED. SUP and INF can be added when the breast is large. So each case had 4 to 8 volumes of data. The quality control standard recommended by the GE ABUS use manual was used for quality control, and the volume data with lesions were collected. In this study, two doctors (XW and YD) with more than 5 years of experience in ABUS were used for quality control.

Two senior radiologists (ZS and CL, with 10 and 15 years of experience in breast ultrasound diagnosis) confirmed all lesions regarding pathological or follow-up results. Cases with differences were judged by another senior radiologist (ZS, with 25 years of experience in breast ultrasound diagnosis).

### Methods and Data Annotation

**Figure 2:**
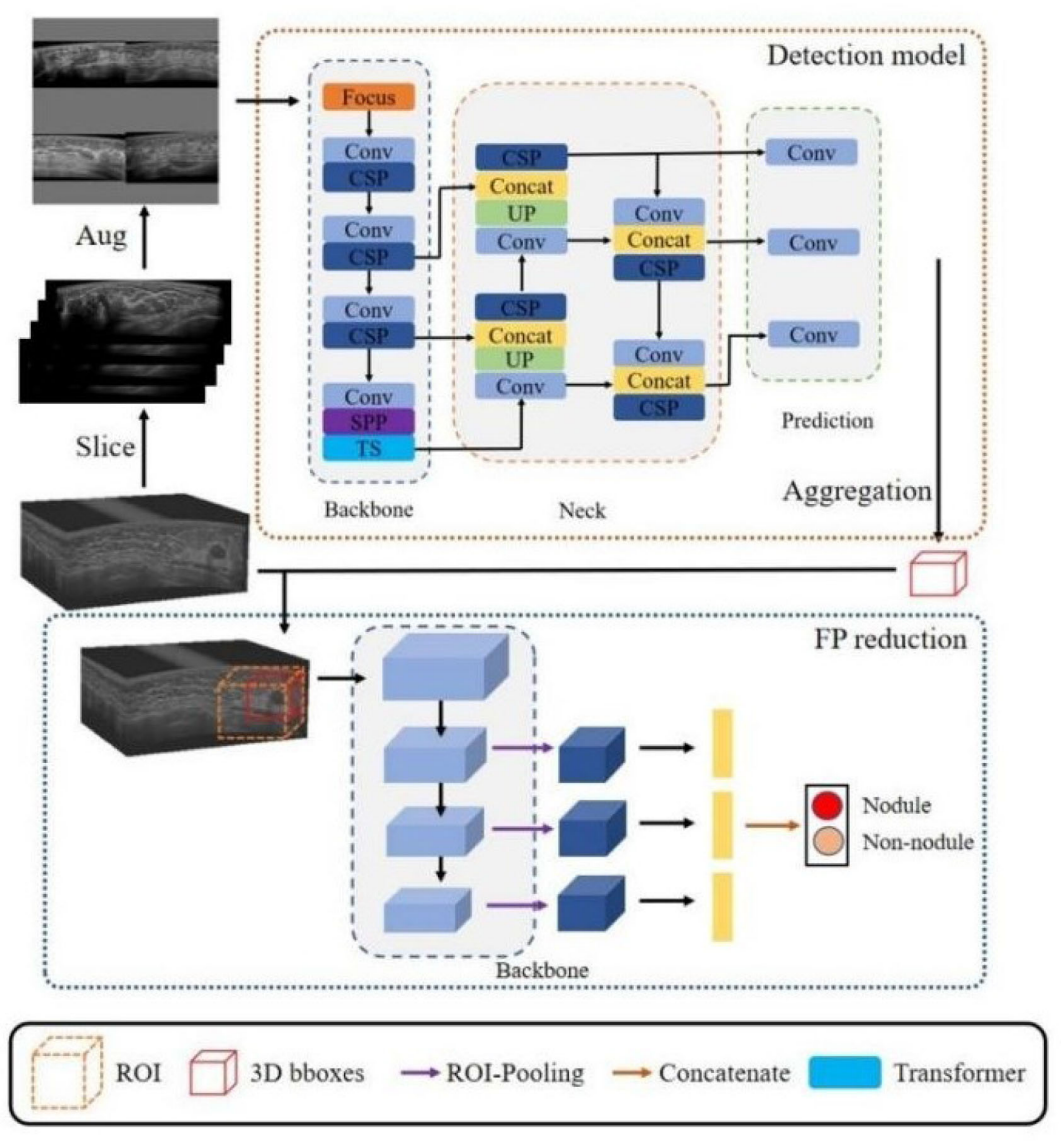
The detection network architecture of our proposed framework. It included a detection model and a three-dimensional false positive reduction model. The ABUS volume was sliced along the cross-section in the detection model training to obtain a two-dimensional image. Four training set images were randomly selected in each study. The improved Mosaic data enhancement method was input into the network for training to develop the three-dimensional false positive reduction model. This model took the lesion as the center, cut the tumour region, inputted it into the three-dimensional classification network, and classified the multi-scale features of the classification network after ROI pooling. In the stage of network reasoning, the slice data of volume are input into the network in turn, and the detection frames of adjacent slices are combined through NMS to obtain a three-dimensional detection frame. According to the three-dimensional detection frame, the ROI area was cut from the original volume data and inputted into the false positive reduction network to obtain the probability that the location was a lesion.

The algorithm (Volume-Breast Ultrasound Intelligent Lesion Detection System, V-BUILDS) was confirmed to have a data enhancement strategy of Mosaic data enhancement ratio of 0.5 and a mixed data enhancement ratio of 0, which was based on Yolo V5. It adopts WBF for model fusion, adds the detection model of the transformer encoder module method, and the algorithm obtained the 3D RESNET reducing false-positive method to detect, verify, and compare the internal and external data. The YOLOv5 classifiers were trained using the open-source Python library Image AI. In the development of the algorithm, the training and testing set were applied to the training and testing of this algorithm. The flowchart is shown in **Figure 2**.

In this study, PAIR was used to annotate ABUS images (PAIR was a multifunctional labeling software developed by the Medical Ultrasonic Image Computing Lab Music of the medical department of Shenzhen University based on C++). The pair has the advantages of supporting multiple data formats, labeling tasks, custom feature attributes, integrating deep learning semi-automatic labeling, and ensuring data security.

### Equipment and Computation Platform

All images were acquired by INVENIA ABUS 1.0 (model 5500-4400-01, GE Healthcare, USA), using a C15-6 × W arc probe with a central frequency of 10 MHz. The examination depth was adjusted according to the size of the breast volume of the patient. The pixel size of the ABUS images was 0.27 × 0.27 × 0.5 mm.

The proposed method was implemented on an NVIDIA RT × 2080TiCPU, Intel(R) × eon(R) Silver 4210 CPU, PyTorch1. 7.0. The model construction of fast RCNN, Retinanet, and Fcos was carried out on the detectron2 framework.

### Classification Performance Evaluation and Statistical Analysis

To evaluate the performance of the breast lesion detection model, accuracy (ACC), sensitivity (SEN), specificity (SPE), false positive (FPS), negative–positive (NPS), positive predictive value (PPV), negative predictive value (NPV), and Youden index (Yi) were evaluated by F1 score (F1). An independent sample t-test was used for inter-group comparison, and the rank-sum test (Mann–Whitney U test) was used for those who did not obey normal distribution or uneven variance. Inspection-level α = 0.05 (normality test, α = 0.10).

Additionally, this study also used the FROC curve to study the relationship between model sensitivity and false-positive ratio, in which vertical sitting represents sensitivity, and the abscissa represents the ratio of the number of false-positive lesions to the number of true positive lesions.

## Results

### Pathology and Follow-Up Results of Different Data Sets

Each data set contained multiple pathological types of lesions confirmed by pathology. As shown in **Table 1**, invasive carcinoma (non-special type) is the most common breast malignant tumor, while fibroadenoma is the most common benign breast tumor. Most lesions were benign and confirmed after more than 2 years of follow-up.

**Table 1:** Pathology and follow-up results of different datasets.

	Pathology or follow-up	Training set (n = 3,883)	testing set (n = 912)	IVD (N = 1,178)	EVD (N = 1,936)
Malignant	invasive carcinoma (non-special type)(B5)	196	46	143	51
	invasive lobular carcinoma(B5)	9	3	3	1
	Ductal carcinoma in situ(B3)	11	7	13	4
	Other types of breast cancer	5	1	2	1
benign	papilloma	24	9	6	2
	Fibroadenoma	413	153	71	52
	hyperplasia	71	15	12	9
	cyst	57	11	7	4
	Other	5	2	4	1
	More then 2-years follow-up	3,092	695	917	1,811

### Lesion Detection in Internal and External Validation Data Based on Different False Positive Values

**Figure 3:**
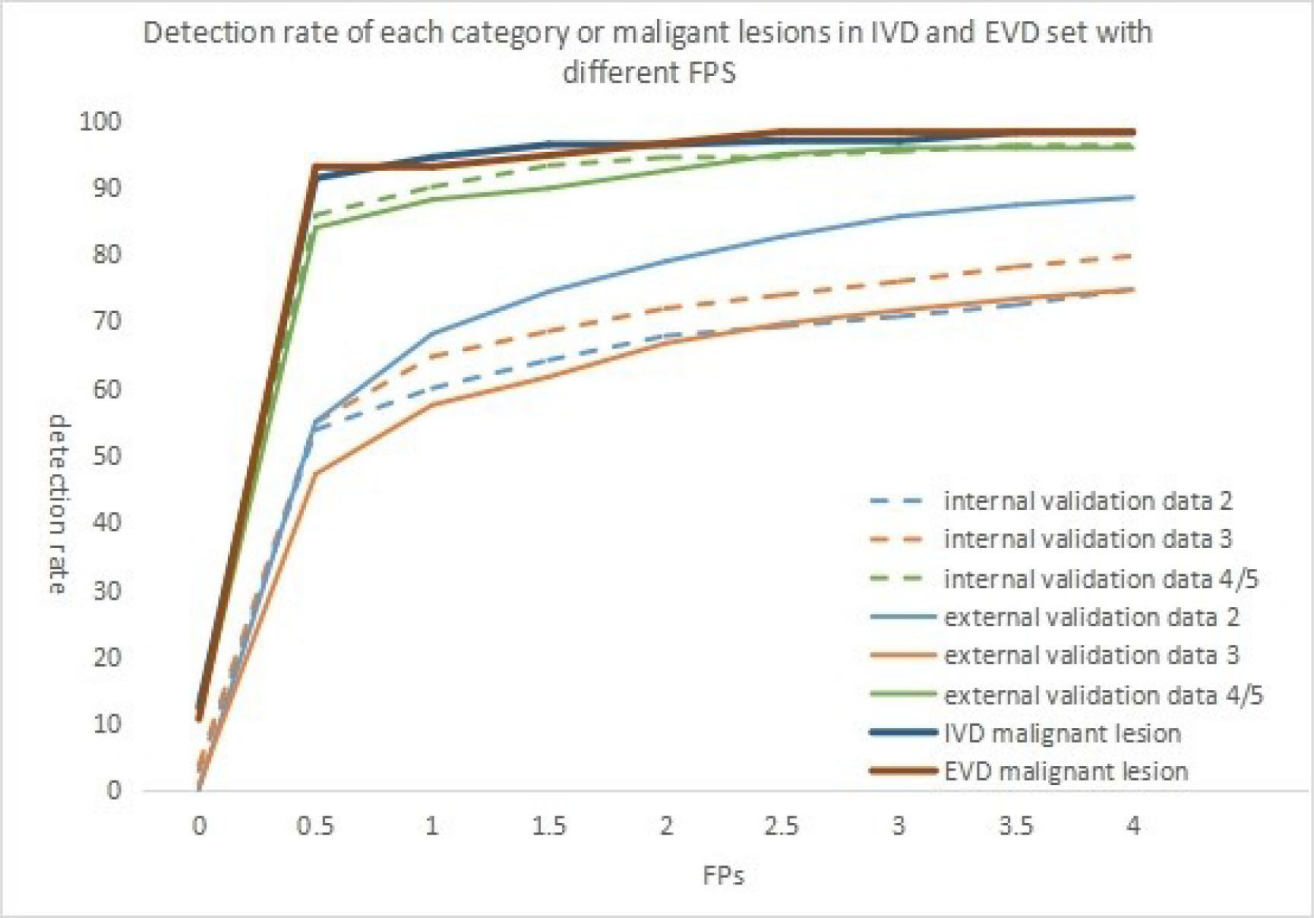
When false positives(FPS) are allowed in different frames, the detection rates of different BI-RADS categories of lesions in different validation sets were shown in the figure. The detection rate of malignant lesion in different validation set was although shown in the figure.

As shown in **Table 2**, the sensitivity indicators of different categories of data to false-positive values were analyzed based on the comparison of internal and external validation data. The overall detection rate of the algorithm was as high as 78.1 and 71.2% in the internal and external validation sets. The study showed that 0.5 false-positive values per frame were susceptible to all categories of data, whether internal or external validation data. With the increase in the number of false positives allowed per frame, the detection sensitivity of each lesion also increased slightly. However, the sensitivity was 93.2% for detecting category 4/5 lesions in the internal validation set when 1.5 false positives were allowed per frame. Moreover, when 4 false positives were allowed per frame, the sensitivity was 96.5%. In the external validation set, the detection performance of category 4/5 lesions was similar. When 3 false positives were allowed per frame, it reached its highest value, and the sensitivity was 95.8%. However, the detection of category 2 and 3 lesions in the internal set and category 3 lesions in the internal and external set failed to reach 80%. With the increase in the number of the false positives allowed per frame, the detection sensitivity increased slightly. The detection rate of BI-RADS 4/5 lesions was higher than that of BI-RADS 3 or 2 (96.5% vs 79.7% vs 74.7% internal, 95.8% vs 74.7% vs 88.4% external) (*P <*0.001). This relationship could be more visually expressed in **Figure 3**.

**Table 2:** When false positives (FPS) are allowed in different frames, the detection rates of different BI-RADS categories of lesions in different validation sets (IVD and EVD) were shown.

	Detection rate (%) of different false positives (FPS)								
	0	0.5	1	1.5	2	2.5	3	3.5	4
IVD category 2*	3	53.8	60	64.2	67.8	69.2	70.7	72.4	74.7
IVD category 3*	3.8	54.9	64.7	68.5	71.9	73.9	75.9	78.1	79.7
IVD category 4/5	13.3	85.8	90	93.2	94.4	94.7	95.3	96.2	96.5
EVD category 2#	0.2	55	68.1	74.4	78.9	82.6	85.6	87.3	88.4
EVD category 3#	0.9	47.2	57.5	61.7	66.7	69.6	71.6	73.3	74.7
EVD category 4/5	11	83.9	88.1	89.8	92.4	94.9	95.8	95.8	95.8

*IVD category 2 VS IVD category 4/5, P < 0.001, IVD category 3 VS IVD category 4/5, P < 0.001, #EVD category 2 VS EVD category 4/5, P < 0.001. # EVD category 3 VS EVD category 4/5, P < 0.001.

### Analysis of Lesion Size and Missed Diagnosis of Internal and External Validation Data

**Figure 4:**
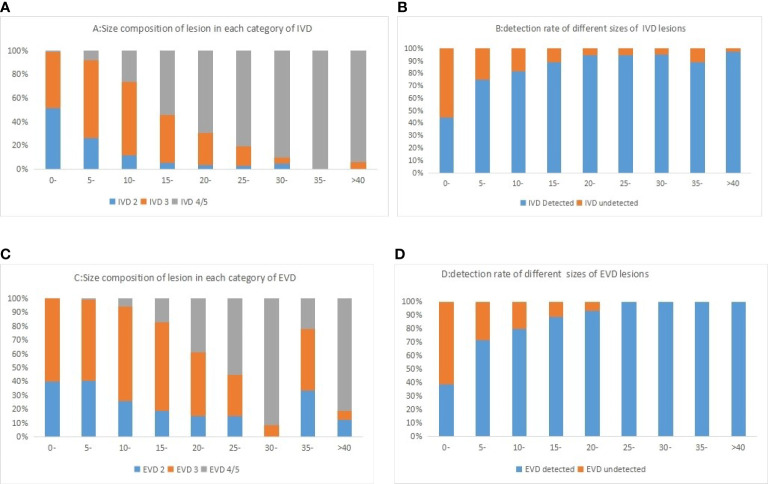
Histogram of different categories of lesions in internal validation data **(A)** and external validation data **(C)** sets and lesions of different sizes was shown in the figure. The detection rate of different sizes of lesion in internal validation data **(B)** and external validation data **(D)** sets was shown in the figure.

In this group of cases (**Table 3**), for the internal validation data set, the non-detection rate of lesions less than 5 mm was 55.6%, and the non-detection rate of 5–10 mm lesions was 25.3%. The non-detection rate of lesions more significant than 10 mm was 12.6%. In the external validation data set, the non-detection rate of lesions less than 5 mm in this algorithm was 61.4%, the non-detection rate of 5–10 mm lesions was 28.8%, and the non-detection rate of lesions more significant than 10 mm was 15.6%.The algorithm could detect more lesions with the increasing nodule size (87.4% in ≥10 mm lesions, but less than 50% in <10 mm). However, there was no difference between the two sets (*P >*0.05). **Figure 4** shows the composition of the size detection relationship of each category.

**Table 3:** In the internal (IVD) and external validation (EVD) sets, the number of possible benign (category 2 or 3) and suspicious malignant lesions (category 4 or 5) of different sizes and the number of detected lesions of different sizes.

	Number of each size										
	0-	5-	10-	15-	20-	25-	30-	35-	>40	*Z*	*P*
IVD category 2 or 3	123	406	240	99	62	24	11	8	27	0.397	0.691
EVD category 2 or 3	264	1021	382	101	36	9	1	7	3		
IDV category 4 or 5	1	28	51	39	29	12	9	1	8	0.708	0.479
EVD category 4 or 5	0	8	23	21	23	11	11	2	13		
IVD detection	55 (44.4)	325 (74.7)	238 (81.8)	123 (89.1)	86 (94.5)	34 (94.4)	19 (95)	8 (88.9)	34 (97.1)	0.177	0.860
EVD detection	102 (38.6)	733 (71.2)	323 (79.8)	108 (88.5)	55 (93.2)	20 (100)	12 (100)	9 (100)	16 (100)		
IVD no detection	69 (55.6)	110 (25.3)	53 (18.2)	15 (10.9)	5 (5.5)	2 (5.6)	1 (5)	1 (11.1)	1 (2.9)	0.489	0.625
EVD no detection	162 (61.4)	296 (28.8)	82 (20.2)	14 (11.5)	4 (6.8)	0 (0)	0 (0)	0 (0)	0 (0)		

*There is no difference between IVD and EVD. But the detection rate of different sizes is different.

### Study on the Detection Rate of Malignant Lesions by Internal and External Validation Data

As shown in **Table 4** and **Figure 3**, the detection rate of malignant lesions was higher than that of category 4/5 lesions (**Table 2**) in the internal validation set. Under the condition of allowing an average of 0.5 false positives per frame, the current detection rate reaches 91.3%. When three false positives per frame were allowed, the detection rate reached 96.9%. The current detection rate reaches 93.0% in the external verification data when the condition of allowing an average of 0.5 false positives per frame is met. When 1.5 false positives per frame were allowed, the detection rate reached 94.7%, and when 2.5 false positives per frame were allowed, the detection rate reached 98.2%.

**Table 4:** The detection rate of malignant lesion in different data set was listed when different false positives (FPS) were allowed.

	Detection rate (%)									
	0	0.5	1	1.5	2	2.5	3	3.5	4	*P*
Malignant in IVD n=161	12.4	91.3	94.4	96.3	96.3	96.9	96.9	98.1	98.1	>0.05
Malignant in EVD n=57	10.7	93.0	93.0	94.7	96.5	98.2	98.2	98.2	98.2	

## Discussion

To our knowledge, this is the first study to develop the lesion detection algorithm based on ABUS volume data and evaluate its internal and external detection efficiency. In recent years, ABUS has become a popular imaging modality in breast cancer detection and diagnosis because ABUS improves the sensitivity of dense breast detection and can be successfully applied to the visualization and characterization of breast lesions (24, 25). Therefore, the analysis of ABUS images has attracted the attention of more and more attention of radiologists and researchers (9). This study applied the deep learning algorithm (V-BUILDS) to the completed model training and 3D false positive reduction network. Based on the ABUS data, the false-positive allowable values of different frames were externally verified for the data from multiple centers. The differences between internal and external verification and the differences in various indicators such as lesion detection efficiency and accuracy were compared and analyzed. The research showed that this algorithm had high detection efficiency for different categories of lesions, and the total detection efficiency for all lesions was slightly lower. However, it had a high detection efficiency for category 4 and 5 lesions, especially malignant lesions. The detection rate for category 4 and 5 lesions differed from that of category 2 or 3 in each dataset (*P* <0.001). The detection rate can reach 0.963 when 1.5 false positives per frame are allowed in the internal verification set.

Some cases are difficult to diagnose because of the characteristics of low signal-to-noise ratio, severe artifacts, blurred margin, and the height change of the shape (9), the small volume of the lesion, and the insufficient number of images. Such lesions may occur in different categories of lesions. However, it is difficult to compare with the surrounding tissues in the detection process, which can easily lead to missed detection or misjudgment. These conditions also occurred in lung lesions detected by CNN (26–28). In this study, when the lesion diameter was less than 10 mm, the detection rate of the lesion was low. However, when the diameter of the lesion was greater than 10 mm, the detection rate of the lesion increased significantly with the increase in the diameter of the lesion. These results were consistent with those reported in the literature (29). It is important to point out that the small lesion is usually the early stage of cancer or a benign lesion. Furthermore, it was difficult to detect with all kinds of images. There were a certain proportion of large undetected and some medium-sized lesions in the external validation data. After analyzing the causes, it was found that the edge of the lesion was blurred, the lesion (a benign lesion) was too large (almost occupying the whole breast), and the lesion scope was uneven and resembled the echo of normal gland tissue. The lesion image (pathological result: malignant lesion) was a non-mass breast lesion with an unclear boundary between the scope and surrounding tissues, heterogeneous echo, ductal hypoechoic, or only localized hypoechoic with distortion of surrounding structures.

In the external validation, the detection of category 3 lesions was always less than that of category 2 lesions. The possible reason was that category 2 lesions were mainly manifested as breast cysts (30, 31), so they had a high detection rate. The hyperplasia lesion in category 3 belonged to uncertain lesions without a clear margin (32). The detection of this lesion needs to be determined by normal glands. When there are numerous hyperplasia lesions in the dataset, it is difficult to identify them by an artificial intelligence algorithm. In this study, there was no statistical difference in detecting malignant lesions in different data sets. The BUILDS achieved high detection sensitivity of 91.3% (IVD) and 93.0% (EVD) at 0.5 false positives per scan. The detection rate in IVD was 98.1% at 3.5 false positives per scan, and the detection rate in EVD was 98.2% at 2.5 false positives per scan. Compared to the reported performance of recently published studies on different datasets, this result was comparable to the numbers reported in a recent multi-view convolutional network study (33) and was much better than the recently published 3D CNN lung lesion detection work (34).

In this group of cases, to ensure that the training and testing set data were widely representative, the included cases were universal and often accompanied by multiple lesions. At the same time, there was the coexistence of benign and malignant lesions, which included numerous hyperplasia lesions. The detection rate of type 2 and 3 lesions in this study was lower, but it was significantly higher than that in previous studies (32, 35–37).

There were several limitations to this study. Firstly, there were some limitations in the amount of research data and some differences in image quality in this multi-center retrospective study. Secondly, the different locations of the lesion may affect the detection of the lesion. When the lesion was located at the edge of the image, it may lead to false judgment due to insufficient local pressure or incomplete display of the lesion. Moreover, if the lesion was located behind the nipple, the lesion was often unclear or incomplete because of the acoustic shadow of the nipple. Additionally, some lesions in this group were too small and lacked pixels, which will also lead to detection difficulties.

This algorithm showed good detection efficiency in internal and external validation, especially for category 4/5 lesions and malignant lesions. However, there are still some deficiencies in detecting category 2 and 3 lesions and lesions smaller than 10 mm. This study showed that this algorithm could be an effective auxiliary tool for lesion detection in ABUS.

## Data Availability Statement

The raw data supporting the conclusions of this article will be made available by the authors, without undue reservation.

## Author Contributions

JXZ: Experimental design, organization and implementation, and thesis writing. XT: Algorithm design and verification and literature novelty search. YHJ: Image annotation and data collection. XXW: Image annotation and data collection. DY: Image annotation and data collection. WX: Image annotation and data collection. SLZ: Image annotation and statistical analysis. LC: Image annotation and experimental verification. LPL: Project acquisition and guide implementation. DN: Algorithm guidance. All authors listed have made a substantial, direct, and intellectual contribution to the work and approved it for publication.

## Conflict of Interest

The authors declare that the research was conducted in the absence of any commercial or financial relationships that could be construed as a potential conflict of interest.

## Publisher’s Note

All claims expressed in this article are solely those of the authors and do not necessarily represent those of their affiliated organizations, or those of the publisher, the editors and the reviewers. Any product that may be evaluated in this article, or claim that may be made by its manufacturer, is not guaranteed or endorsed by the publisher.
